# Significance of 8-OHdG Expression as a Predictor of Survival in Colorectal Cancer

**DOI:** 10.3390/cancers15184613

**Published:** 2023-09-18

**Authors:** Myunghee Kang, Soyeon Jeong, Sungjin Park, Seungyoon Nam, Jun-Won Chung, Kyoung Oh Kim, Jungsuk An, Jung Ho Kim

**Affiliations:** 1 Department of Pathology, Gachon University Gil Medical Center, College of Medicine, Gachon University, Incheon 21565, Republic of Korea; kangmh@gilhospital.com; 2 Gachon Biomedical Convergence Institute, Gachon University Gil Medical Center, College of Medicine, Gachon University, Incheon 21565, Republic of Korea; jensyj85@gmail.com; 3 Department of Health Sciences and Technology, Gachon Advanced Institute for Health Sciences and Technology (GAIHST), Gachon University, Incheon 21999, Republic of Korea; oscar.park@gmail.com (S.P.); nams@gilhospital.com (S.N.); 4 Department of Genome Medicine and Science, AI Convergence Center for Genome Medicine, Gachon Institute of Genome Medicine and Science, Gachon University Gil Medical Center, College of Medicine, Gachon University, Incheon 21565, Republic of Korea; 5 Department of Internal Medicine, Gachon University Gil Medical Center, College of Medicine, Gachon University, Incheon 21565, Republic of Korea; drgreen@gilhospital.com (J.-W.C.); kkoimge@gilhospital.com (K.O.K.); 6 Department of Pathology, Korea University Anam Hospital, Korea University College of Medicine, Seoul 02841, Republic of Korea; 7 Department of Translational-Clinical Medicine, Gachon Advanced Institute for Health Sciences and Technology (GAIHST), Gachon University, Incheon 21999, Republic of Korea

**Keywords:** colorectal cancer, 8-OHdG, biomarker, survival prediction, TNM classification

## Abstract

**Simple Summary:**

Although oxidative stress regulates essential signaling pathways, oxidative DNA damage causes cancer initiation and progression. Given that the relationship between oxidative stress and colorectal cancer (CRC) remains poorly understood, we performed immunohistochemistry to detect 8-hydroxy-2′ deoxyguanosine (8-OHdG) in 564 patients with CRC and conducted survival analysis based on the pathological stage to identify novel biomarkers. We found that low 8-OHdG levels were associated with a poor prognosis. Further, when 8-OHdG expression was combined with the tumor node metastasis stage, if 8-OHdG expression was low in the same stage, the prognosis was poor, suggesting that 8-OHdG may be an essential biomarker of CRC.

**Abstract:**

The incidence of colorectal cancer (CRC) is increasing worldwide. 8-hydroxy-2′-deoxyguanosine (8-OHdG), one of the most prevalent DNA alterations, is known to be upregulated in several carcinomas; however, 8-OHdG has not been used to predict the prognosis of patients with CRC. We aimed to determine 8-OHdG levels in patients with CRC using immunohistochemistry and conducted a survival analysis according to the pathological stage. The 5-year event-free survival (EFS) and disease-specific survival (DSS) hazard ratios (HRs) of the low 8-OHdG subgroup were 1.41 (95% confidence interval (CI): 1.01–1.98, *p* = 0.04) and 1.60 (95% CI: 1.12–2.28, *p* = 0.01), respectively. When tumor node metastasis (TNM) staging and 8-OHdG expression were combined, the 5-year EFS and DSS HRs of patients with CRC with low 8-OHdG expression cancer at the same TNM stage (stage Ⅲ/Ⅳ) were 1.51 (95% CI: 1.02–2.22, *p* = 0.04) and 1.64 (95% CI: 1.09–2.48, *p* = 0.02), respectively, compared to those with high 8-OHdG expression cancer, indicating a poor prognosis. Therefore, low 8-OHdG expression is a significant predictive factor for 5-year EFS and DSS in patients with CRC, and it can serve as an essential biomarker of CRC.

## 1. Introduction

Colorectal cancer (CRC) is the most common malignant tumor of the gastrointestinal tract, and its incidence is increasing [1]. Many studies conducted on various factors that cause CRC [2] have suggested that reactive oxygen species (ROS)-induced DNA damage has a significant effect on carcinogenesis and cancer progression [3].

ROS, also called free radicals, are by-products of respiration and energy production in aerobic organisms; mitochondria are the primary source of ROS [4]. Although ROS are crucial signaling pathway regulators, oxidative stress occurs when ROS production exceeds the cell’s antioxidant capacity [5,6]. Hypoxia, inflammation, and exposure to specific foods and tobacco all enhance extracellular ROS production in the intestinal mucosa in CRC [7]. Free radicals are volatile because of their unpaired electrons which rapidly react with other molecules; they can also directly interact with DNA as well as oxidize lipids and proteins to produce intermediates that react with DNA [8]. During oxidative stress, ROS can cause DNA alterations such as strand damage, pyrimidine and purine base modifications, and sister chromatid exchange [5]. Moreover, ROS can either inactivate tumor suppressor genes or promote proto-oncogene expression [9].

Hydroxyl radicals (·OH) play an important role in DNA oxidation, and numerous forms of oxidized nucleosides have been identified [10]. Guanine is the most vulnerable nucleobase to ROS oxidation. One of the most prevalent DNA alterations is 8-hydroxy-2′-deoxyguanosine (8-OHdG), which results in oxidative damage of 2′-deoxyguanosine [11]. The formation of 8-OHdG in DNA results in a G:C–T:A mispairing mutation, which is associated with tumor development and progression, cellular senescence, and degenerative diseases [12]. 8-OHdG expression in tissues, serum, and urine is upregulated in several carcinomas, including ovarian cancer, breast cancer, prostate cancer, non-small cell lung cancer (NSCLC), esophageal cancer, and CRC [13,14,15,16,17].

It is possible that the increase in 8-OHdG in cancer is related to cancer prognosis. Moreover, 8-OHdG may be associated with clinical factors or be a survival predictor in cancer. However, the impact of 8-OHdG on predicting patient prognosis remains controversial, and there are few reports on its clinical significance in tumors. For example, the survival period is long when the 8-OHdG expression is high in ovarian cancer or NSCLC [18,19], whereas, in triple-negative breast cancer and basal-like breast cancer, the survival period is shorter when the 8-OhdG expression is high [20,21]. Therefore, we aimed to verify the clinical significance of 8-OHdG in CRC in terms of personalized treatment and prevention and its potential as a predictive factor for survival.

In this study, we evaluated the clinicopathologic significance of 8-OHdG expression in patients with CRC through univariate/multivariate analysis. Furthermore, to investigate the potential of 8-OHdG as an independent prognostic factor concerning survival, which is the most crucial factor in oncological outcomes, 8-OHdG levels were determined via immunohistochemistry (IHC) in tissues from patients with CRC, and patient survival was analyzed accordingly.

## 2. Materials and Methods

### 2.1. Patient Population and Clinical Specimens

Patients who underwent CRC surgery at the Gachon University Gil Medical Center between April 2010 and November 2012 were included in this study. Overall, 564 patients with primary CRC who underwent surgery and had preserved paraffin blocks were examined. Patients with recurrent CRC, alterations in the normal intestinal structure of the colon due to previous surgery, a history of chemotherapy or abdominal radiation therapy before CRC surgery, and those previously treated for other cancers were excluded. This study was approved by the Institutional Review Board of the Gachon University Gil Medical Center (GCIRB2023-176).

### 2.2. Definition of Clinicopathologic Factors

The definition of clinicopathologic factors was the same as previously reported [22]. A family history of CRC is associated with the occurrence of CRC in first-degree relatives. Patients who had a smoking history included either those having previously smoked or current smokers. Anemia was marked by a hemoglobin level < 13 g/dL in men and <12 g/dL in women. Aberrant levels of white blood cells (WBCs) were categorized as leukocytosis when the count exceeded 10,000 cells/L, or as leukopenia when the count was under 4000 cells/L. An elevated level of carcinoembryonic antigen (CEA) exceeding 5 ng/mL was an aberrant condition. The classification of CRC was based on the primary tumor’s anatomical location. Tumors in the cecum, ascending colon, or transverse colon extending to the hepatic flexure are usually referred to as right-sided colorectal cancer (RCC). Left-sided colorectal cancer (LCC) includes cancer occurring at the splenic flexure and the regions located distally to the splenic flexure, such as the rectum.

Cancers were categorized as differentiated or undifferentiated based on pathological features that influence CRC differentiation and prognosis. Tumors with well differentiation (WD) and moderate differentiation (MD) were classified as differentiated, and those poorly differentiated (PD) and tumors with signet ring cell and mucinous carcinomas were classified as undifferentiated.

### 2.3. Tissue Microarray (TMA) and IHC

The TMA staining was the same method used in our previous study [22]. Briefly, TNM slides were baked, deparaffinized, and rehydrated to inhibit endogenous peroxidase activity, and then antigens were electrolyzed. After 30 min of preincubation in 10% normal goat serum (#31872, Invitrogen, Danvers, MA, USA) to prevent nonspecific staining, samples were incubated overnight in a humidified container at 4 °C with anti-8-OHdG (1:200, N45.1, monoclonal, Abcam, Cambridge, UK). A non-biotin horseradish peroxidase detection system (Gene Tech, San Francisco, CA, USA) was used to treat tissue slides as instructed.

**Figure 1:**
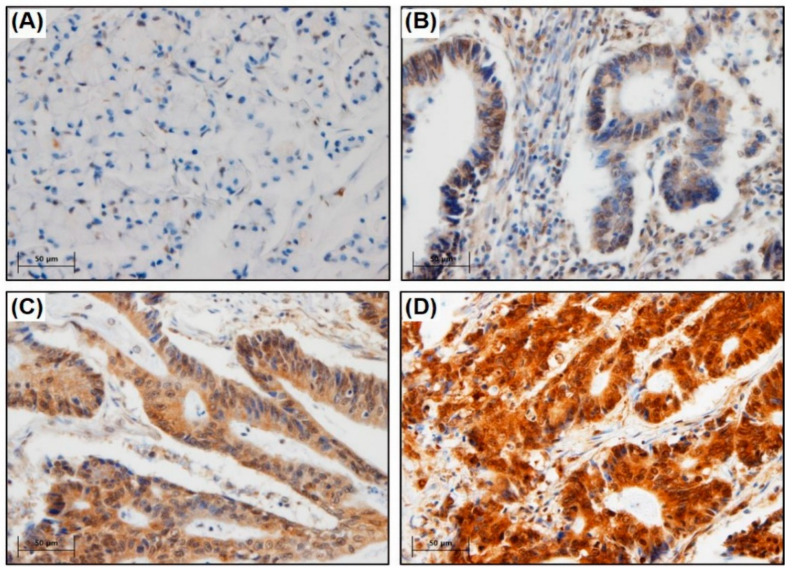
Immunohistochemical staining for 8-OHdG was classified into four groups according to their nuclear intensity in adenocarcinoma cells. (**A**) Score 0 show faint and rare immunostaining; (**B**) Score 1+ show focal and weak immunostaining; (**C**) Score 2+ show moderate and diffuse immunostaining; (**D**) Score 3+ show strong and diffuse immunostaining. Original magnification, 400×; Scale bar, 50 μm.

The degree of 8-OHdG expression was categorized into four classes (0–3). TMA slides were blindly assessed twice by two experienced pathologists (M.K., J.A.). To account for discrepant scores among the pathologists, the slide was re-examined by both pathologists simultaneously using a multi-head microscope. The intensity of 8-OHdG expression was classified via IHC as follows: 0, no staining; 1, faint staining; 2, moderate staining; and 3, strong staining. For expression levels, 0–2 points were denoted as low expression and 3 points as high expression (Figure 1).

### 2.4. Statistical Analysis

Continuous variables were expressed as means ± standard deviation, whereas categorical variables were represented as absolute numbers and percentages. The Chi-square test was used to determine the relationship between 8-OHdG expression and clinical data. This study addressed the survival-related characteristics and survival rates after CRC surgery, focusing on event-free survival (EFS) and disease-specific survival (DSS). Cancer progression, recurrence, and mortality due to CRC were all considered indicators of EFS. DSS was defined as the time until death from CRC after surgery and was considered censored in the event of death from other diseases.

As TNM classification and 8-OHdG expression predicted survival, patients were divided into four groups (Group 1, high 8-OHdG expression [8-OHdG_High_] and TNM classification stages I/II; Group 2, low 8-OHdG expression [8-OHdG_Low_] and TNM classification stages I/II; Group 3, 8-OHdG_High_ and TNM classification stages III/IV; and Group 4, 8-OHdG_Low_ and TNM classification stages III/IV). EFS and DSS according to 8-OHdG expression level were also analyzed, reflecting the TNM stage.

The log-rank test was used to plot survival curves using the Kaplan–Meier method. Cox proportional hazards (CPH) regression was used for survival analysis. SPSS version 22.0 (SPSS Inc., Chicago, IL, USA) was used for statistical analyses. Statistical significance was set at *p* < 0.05 (two-sided). The “forestmodel” package in R version 4.0 was used to calculate the hazard ratios (HR) for each variable obtained using the CPH model.

## 3. Results

### 3.1. Baseline Characteristics

This study included 564 patients with CRC in total. Table 1 shows the baseline characteristics of the patients with CRC participating in the study. The mean age of the 564 patients examined was 64.3 ± 11.4 years, and the mean tumor size was 52.7 ± 23.2 mm. The mean hemoglobin, WBC, and CEA levels were 12.2 ± 2.4 g/dL, 7664.0 ± 3.0 10^3^/μL, and 27.0 ± 303.1 ng/mL, respectively. The most common location of CRC was the rectum, which accounted for 145 (25.7%) cases, and the most common degree of differentiation was MD cancer, which accounted for 476 (84.4%) cases. CRC was classified as follows by TNM: 98 (17.4%) stage I, 206 (36.5%) stage II, 189 (33.5%) stage III, and 71 (12.6%) stage IV.

The clinical significance of 8-OHdG expression is shown in Table 2. 8-OHdG_High_ was identified in 379/564 (67.2%) patients. The results of univariate and multivariate analyses showed that in the 8-OHdG_High_ group, differentiated cancer and undifferentiated cancer were 353/513 (93.1%) and 26/51 (6.9%), respectively, and in the 8-OHdG_Low_ group, differentiated cancer and undifferentiated cancer were 160/513 (86.5%) and 25/51 (13.5%), respectively (*p* = 0.010). This showed that the 8-OHdG_High_ group had statistically significant higher numbers of differentiated cancer compared to the 8-OHdG_Low_ group (*p* = 0.012).

### 3.2. Low 8-OHdG Expression Is Related to Poor Prognosis

**Figure 2:**
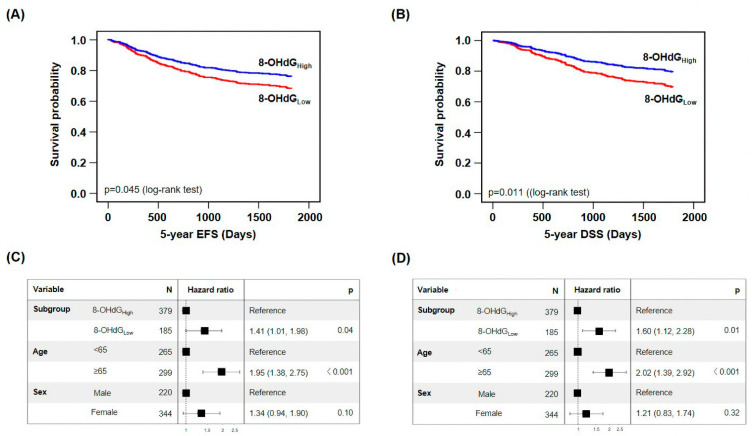
Low 8-OHdG expression is associated with poor prognosis in patients with colorectal cancer (CRC). Event-free survival (EFS) (**A**,**C**) and disease-specific survival (DSS) (**B**,**D**) in patients with CRC based on 8-OHdG expression.

We performed survival analysis to evaluate the association between 8-OHdG expression and 5-year EFS and DSS, which revealed that the 8-OHdG_Low_ survival rate within 5-year EFS was 69.7%, the 8-OHdG_High_ survival rate was 76.8% (*p* = 0.045), and the HR of the 8-OHdG_Low_ group versus the 8-OHdG_High_ group was 1.41 (CPH model adjusted for age and sex, 95% confidence interval [CI]: 1.01–1.98, *p* = 0.04) (Figure 2A,C). The median 5-year EFS for the 8-OHdG_Low_ and 8-OHdG_High_ groups was 1421.3 and 1550.2 days, respectively.

Furthermore, the 8-OHdG_Low_ with the 5-year DSS was 71.9%, which was lower than the 8-OHdG_High_ (80.5%, *p* = 0.011), and the HR of the 8-OHdG_Low_ group versus the 8-OHdG_High_ group was 1.60 in the multivariate model (CPH model adjusted for age and sex, 95% CI: 1.12–2.28, *p* = 0.01) (Figure 2B,D). The median 5-year DSS for the 8-OHdG_Low_ and 8-OHdG_High_ groups was 1467.8 days and 1622.5 days, respectively. These data indicate that low 8-OHdG expression is associated with poor prognosis.

### 3.3. Low Expression of 8-OHdG at Advanced Stage Is Associated with Poor Prognosis

To evaluate the impact of 8-OHdG expression more precisely on the prognosis of patients with CRC, survival analysis was performed wherein the 8-OHdG expression level and TNM stage were combined. First, we stratified the patient population into four subgroups based on 8-OHdG expression levels and TNM stage: Group 1 (8-OHdG_High_ and TNM stages I/II, n = 208), Group 2 (8-OHdG_Low_ and TNM stages I/II, n = 96), Group 3 (8-OHdG_High_ and TNM stages III/IV, n = 171), and Group 4 (8-OHdG_Low_ and TNM stages III/IV, n = 89). 

**Figure 3:**
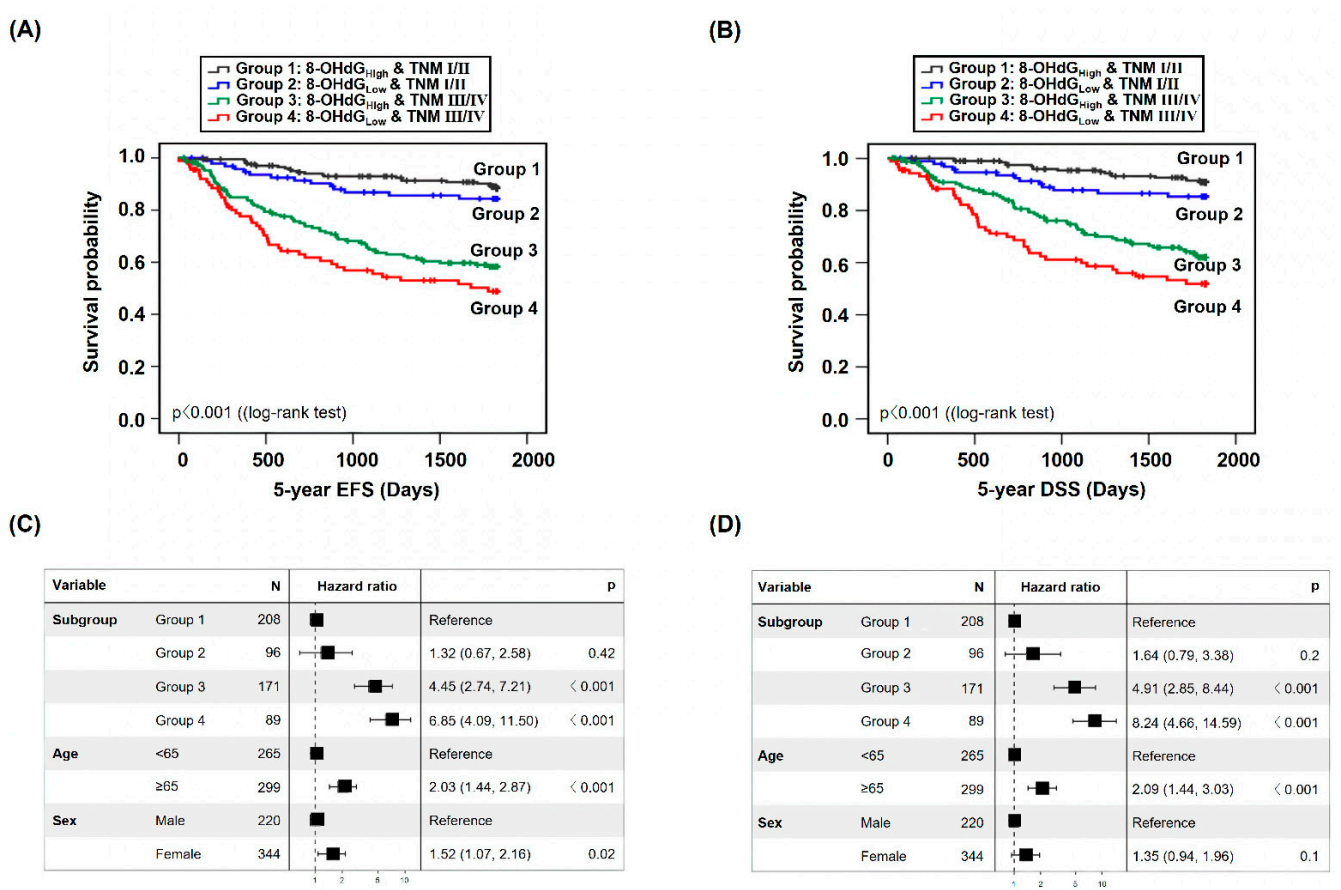
Survival analysis of 8-OHdG expression in patients with CRC. Survival analysis of EFS (**A**,**C**) and DSS (**B**,**D**) based on the combination of 8-OHdG expression and TNM stage.

Compared with Group 1, the HR of 5-year EFS was 1.32 (CPH model adjusted for age and sex, 95% CI: 0.67–2.58, *p* = 0.42) in Group 2, 4.45 (CPH model adjusted for age and sex, 95% CI: 2.74–7.21, *p* < 0.001) in Group 3, and 6.85 (CPH model adjusted for age and sex, 95% CI: 4.09–11.50, *p* < 0.001) in Group 4 (Figure 3A,C).

The HR of DSS was 1.64 (CPH model adjusted for age and sex, 95% CI: 0.79–3.38, *p* = 0.2) in Group 2 vs. Group 1, 4.91 (CPH model adjusted for age and sex, 95% CI: 2.85–8.44, *p* < 0.001) in Group 3 vs. Group 1, and 8.24 (CPH model adjusted for age and sex, 95% CI: 4.66–14.59, *p* < 0.001) in Group 4 vs. Group 1 (Figure 3B,D).

**Figure 4:**
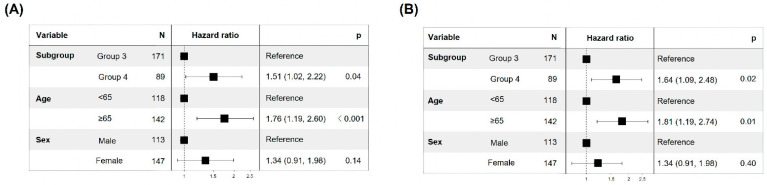
Low 8-OHdG expression has a poorer prognosis than high 8-OHdG expression at the same stage. Survival analysis of EFS (**A**) and DSS (**B**) based on the combination of 8-OHdG expression and TNM stage.

In patients at the same stage (TNM classification stage III/IV), the HR of EFS and DSS in the 8-OHdG_Low_ group was 1.51 (CPH model adjusted for age and sex, 95% CI: 1.02–2.22, *p* = 0.04) and 1.64 (CPH model adjusted for age and sex, 95% CI: 1.09–2.48, *p* = 0.02), respectively, compared to the 8-OHdG_High_ group (Figure 4A,B). These data suggest that low 8-OHdG expression in patients with same-stage (advanced-stage) CRC is associated with a poor prognosis.

## 4. Discussion

The incidence and mortality rates of CRC are continuously increasing [1]. The pathological stage of most malignancies, including CRC, predicts the prognosis of patients. However, because patients with the same pathological stage can exhibit different clinical outcomes, new potential biomarkers for accurately predicting the prognosis of patients with cancer are urgently required.

Oxidative DNA damage is linked to various diseases and is essential in the initiation and progression of cancers, including CRC [23,24]. However, ROS are extremely unstable, making exact measurements impossible. Stable metabolites, particularly 8-OHdG, are indicators of oxidative stress and cancer, and are frequently used to assess the degree of DNA damage in patients exposed to carcinogens such as cigarettes and asbestos [25]. Nevertheless, the exact relationship between oxidative stress and CRC remains unclear, and there are few studies on the subject, particularly in clinical settings. Therefore, this study is significant in that it revealed that 8-OHdG can be applied in the clinical setting by identifying 8-OHdG’s clinical importance and confirming its potential as a survival predictor.

According to our findings, 8-OHdG was a significant independent factor identified in multivariate analysis. In other words, 8-OHdG expression was not influenced by other factors including TNM stage exception differentiation degree. When 8-OHdG expression was low, the rate of undifferentiated cancer was higher than the rate of differentiated cancer, which is similar to the result in breast cancer [26,27]. Differentiation is strongly correlated with TP53, and 8-OHdG is also linked to p53. TP53 is a well-known tumor suppressor protein essential for protecting DNA integrity [28]. In addition, ROS induces modification of the G:C ration of TP53 in lung and liver cancer, and the TP53 mutation is mainly observed in poorly differentiated cancer, such as ovarian cancer [29,30,31,32]. Thus, the close relationship between the low expression of 8-OHdG and poor differentiation may be due to genetic alterations in TP53 caused by oxidative stress, and further studies are needed.

The 8-OHdG expression in cancer is higher than normal and increased 8-OHdG expression has been associated with a poor prognosis in ovarian cancer, hepatocellular carcinoma, and melanoma [18,33,34,35]. Our IHC results also revealed increased 8-OHdG expression in patients with CRC, but the prognosis was poorer when the 8-OHdG expression was decreased. Additionally, similar to our findings, poor prognosis was associated with low 8-OHdG expression in triple-negative and basal-like breast cancer [20,21].

There are several potential mechanisms by which low expression of 8-OHdG may lead to a poor prognosis. First, nuclear factor erythroid-2-related factor 2 (Nrf2), one of the antioxidant enzymes, regulates antioxidant pathways. As a defense against ROS production in tumors, many antioxidant mechanisms are activated that reduce 8-OHdG expression levels in tumor tissues by preventing ROS from interacting with DNA or directly removing 8-OHdG [20,36]. Nrf2 is released from the Keap1/Nrf2 complex and migrates to and accumulates in the nucleus. Subsequently, the number of Nrf2-induced redox state-regulating enzymes increase [37]. Antioxidant enzymes produced through this series of processes can reduce 8-OHdG expression and offset the negative effects of oxidative stress, thereby promoting cancer progression and potential metastasis. Second, base-excision repair (BER) enzymes regulate 8-OHdG expression. BERs, such as 8-oxoguanine glycosylase (OGG1) and MutY homolog, and nucleotide pool sanitizing enzymes such as MutT homolog 1, generally repair DNA damage [38]. In particular, OGG1 can regulate the expression of 8-OHdG. Normally, 8-OHdG expression is regulated by hydrolysis or the cleavage of incorrectly bonded nucleotides by OGG1 [26]; impaired OGG1 activity damages DNA and cannot cleave guanosine, thus protecting the integrity of 8-OHdG, resulting in cancer development and a poor patient prognosis [39]. These mechanisms may explain the poor prognosis of patients with low 8-OHdG expression; however, additional studies are required.

So far, prognosis has been predicted according to the expression of 8-OHdG in various cancers, and the results are diverse. Some research results are similar to ours, while others are different. Several factors can lead to different results depending on 8-OHdG expression. First, there is a difference in the detection method of 8-OHdG. Many studies have used TCGA data, urine, and serum samples to determine 8-OHdG expression [33,40,41]. In one study, survival analysis was performed based on 8-OHdG expression using enzyme-linked immunosorbent assay (ELISA) and quantitative IHC (qIHC); a poor prognosis was associated with higher 8-OHdG expression in ELISA and lower 8-OHdG expression in qIHC [20]. Second, because IHC was conducted on different organs and races, the results may vary depending on genetic variables. Other studies were conducted on breast cancer, NSCLC, and ovarian cancer in Europeans and Japanese people [18,19,20]. Third, the number of samples studied is also important. Most studies on 8-OHdG were conducted on less than 200 patients. However, our study is reliable since it included clinical data from more than 500 Korean patients with CRC. For these reasons, it is possible that the differences in the results were due to the different detection methods used for assessing 8-OHdG expression, the analysis of different types of cohorts, and the number of samples used in the research.

TNM staging has long been used in clinical practice for treatment and prognosis prediction. However, TNM staging alone is insufficient for personalized medicine. Therefore, the development of various biomarkers with clinical significance is necessary. In other words, combining TNM stage, which is supported by many years of experience, and various biomarkers would allow for more precise patient treatment.

In this study, 8-OHdG was found to be a prognostic predictor for patients with CRC. Additionally, when combined with TNM stage, 8-OHdG was able to predict patient prognosis more precisely. Furthermore, in order to be promptly implemented in clinical practice, the analysis method must be simple and affordable. Because the IHC used in this study is thought to be suitable for clinical use. In this regard, there is a need for the development of various clinically significant biomarkers, such as 8-OHdG, that can be evaluated in a simple and affordable manner. In light of the advantages of biomarkers, once they are implemented in combination with TNM staging, personalized medicine will become possible.

## 5. Conclusions

**Figure 5:**
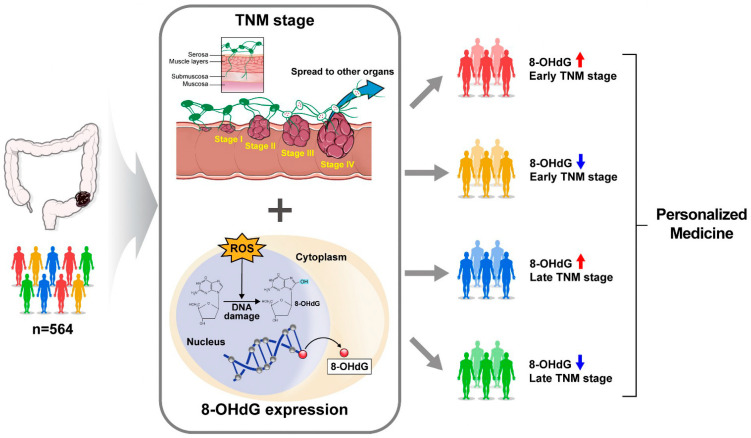
Schematic representation of personalized medicine based on the 8-OHdG expression and TNM stage. Red arrow: high expression; Blue arrow: low expression.

In conclusion, 8-OHdG expression was independent of the TNM stage in our cohort, but considerably related to the degree of differentiation. Notably, when TNM stage and 8-OHdG levels were combined in the survival analysis, a more accurate prognosis prediction was possible. The lower the expression of 8-OHdG, the poorer the prognosis, and the lower the expression of 8-OHdG at the same TNM stage (stages III/IV), the worse the survival rate. These findings suggest the potential of 8-OHdG as a reliable marker for clinical as well as survival prognostic factors (Figure 5).

## Figures and Tables

**Table 1:** Baseline characteristics of participants.

	Total (n = 564)
Age	64.3 ± 11.4
Sex	
Male	344 (61.0%)
Female	220 (39.0%)
Laboratory findings	
Hemoglobin (g/dL)	12.2 ± 2.4
WBC (10^3^/μL)	7664.0 ± 3.0
CEA (ng/mL)	27.0 ± 303.1
Pathology	
Size (mm)	52.7 ± 23.2
Location	
Cecum	11 (2.0%)
Ascending colon	91 (16.1%)
Hepatic flexure	8 (1.4%)
Transverse colon	37 (6.6%)
Splenic flexure	4 (0.7%)
Descending colon	18 (3.2%)
Sigmoid-descending	5 (0.9%)
Sigmoid colon	141 (25.0%)
Rectosigmoid colon	104 (18.4%)
Rectum	145 (25.7%)
Differentiation	
WD	37 (6.6%)
MD	476 (84.4%)
PD	24 (4.3%)
Mucinous	24 (4.3%)
SRC	2 (0.4%)
TNM stage	
Ⅰ	98 (17.4%)
Ⅱ	206 (36.5%)
Ⅲ	189 (33.5%)
Ⅳ	71 (12.6%)

CEA, carcinoembryonic antigen; MD, moderately differentiated; PD, poorly differentiated; SRC, signet ring cell; TNM, tumor node metastasis; WBC, white blood cell; WD, well differentiated.

**Table 2:** Clinicopathologic significance of 8-OHdG expression.

Variables		Univariate			Multivariate	
	Total (n = 564)	8-OHdG (n = 185), Low	8-OHdG (n = 379), High	*p*	OR (CI)	*p*
Age (years)				0.464		0.464
< 65	265 (47.0%)	91 (49.2%)	174 (45.9%)		1 (Reference)	
≥65	299 (53.0%)	94 (50.8%)	205 (54.1%)		1.414 (0.082–1.622)	
Sex				0.133		0.082
Male	344 (61.0%)	121 (65.4%)	223 (58.8%)		1 (Reference)	
Female	220 (39.0%)	64 (34.6%)	156 (41.2%)		1.420 (0.956–2.109)	
Diabetes mellitus				0.672		0.553
No	464 (82.3%)	154 (83.2%)	310 (81.8%)		1 (Reference)	
Yes	100 (17.7%)	31 (16.8%)	69 (18.2%)		1.159 (0.712–1.887)	
Smoking				0.666		0.877
No	433 (76.8%)	140 (75.7%)	293 (77.3%)		1 (Reference)	
Yes	131 (23.2%)	45 (24.3%)	86 (22.7%)		0.965 (0.616–1.513)	
Family history				0.371		0.408
No	543 (96.3%)	180 (97.3%)	363 (95.8%)		1 (Reference)	
Yes	21 (3.7%)	5 (2.7%)	16 (4.2%)		1.546 (0.551–4.341)	
Anemia				0.247		0.215
No	291 (51.6%)	89 (48.1%)	202 (53.3%)		1 (Reference)	
Yes	273 (48.4%)	96 (51.9%)	177 (46.7%)		0.785 (0.535–1.273)	
WBC counts				0.232		0.316
Normal	478 (84.8%)	152 (82.2%)	326 (86.0%)		1 (Reference)	
Abnormal	86 (15.2%)	33 (17.8%)	53 (14.0%)		0.777 (0.474–1.273)	
Serum CEA				0.237		0.379
Normal	417 (73.9%)	131 (70.8%)	286 (75.5%)		1 (Reference)	
Abnormal	147 (26.1%)	54 (29.2%)	93 (24.5%)		0.828 (0.543–1.261)	
Tumor location				0.617		0.901
LCC	416 (73.8%)	134 (72.4%)	282 (74.4%)		1 (Reference)	
RCC	148 (26.2%)	51 (27.6%)	97 (25.6%)		0.973 (0.636–1.490)	
Tumor				0.010		0.012
Differentiation	513 (91.0%)	160 (86.5%)	353 (93.1%)		1 (Reference)	
Undifferentiation	51 (9.0%)	25 (13.5%)	26 (6.9%)		0.461 (0.252–0.844)	
TNM stage				0.504		0.985
Ⅰ/Ⅱ	304 (53.9%)	96 (51.9%)	208 (54.9%)		1 (Reference)	
Ⅲ/Ⅳ	260 (46.1%)	89 (48.1%)	171 (45.1%)		0.973 (0.636–1.490)	

CEA, carcinoembryonic antigen; CI, confidence interval; OR, odds ratio; LCC, left-sided colorectal cancer; RCC, right-sided colorectal cancer; TNM, tumor node metastasis; WBC, white blood cell.

## Data Availability

Data are available upon reasonable request to the authors.
